# A132 ACHALASIA FOLLOWING A SARS-COV-2 INFECTION: A CASE REPORT

**DOI:** 10.1093/jcag/gwab049.131

**Published:** 2022-02-21

**Authors:** E Zeidan, E Desilets, M Bouin, T Maniere

**Affiliations:** 1 Medicine, Universite de Sherbrooke, Sherbrooke, QC, Canada; 2 Universite de Montreal, Montreal, QC, Canada

## Abstract

**Background:**

SARS-CoV-2 is a novel virus currently causing a major pandemic. Recent studies have shown that SARS-CoV-2 has an affinity to ACE2 receptors which are present in the olfactory epithelium as well as the pulmonary and gastrointestinal (GI) systems. It is stipulated that these receptors are responsible for the entry of the virus into cells explaining the respiratory and GI symptoms of this infection. The anosmia related to COVID-19 is likely due to an aberrant immune response to the virus rather than direct damage to the olfactory epithelium. Similarly, achalasia is also believed to result from an aberrant immune response through which there is degeneration of inhibitory neurons in the myenteric plexus. In this case report, we present the first case of achalasia thought to be linked to a SARS-CoV-2 infection in contemporary literature.

**Aims:**

We hypothesize that SARS-CoV-2 caused an aberrant immune response in our patient, leading him to develop achalasia. We hope to expose clinicians to this potential complication of COVID-19, allowing them to consider it sooner in their differential.

**Methods:**

The patient’s file was reviewed to extract the relevant information. A second gastroenterologist’s opinion was obtained on the manometry to confirm the diagnosis of type III achalasia.

**Results:**

A 61-year-old man with a medical history of myasthenia gravis, chronic obstructive pulmonary disease, hypothyroidism, and chronic renal failure was treated in our establishment for a SARS-CoV-2 pneumonia. He experienced new severe dysphagic symptoms, regurgitations, and lost 10 pounds during his hospitalization. He also developed anosmia and diarrhea. A CT-scan of his neck and thorax showed no extrinsic compression of his esophagus. No neoplasm was found on his gastroscopy. A barium study was then conducted showing a stagnation of contrast in the distal third of his esophagus. No relaxation of the inferior gastroesophageal sphincter was noted. A manometry was then performed and confirmed a type III achalasia. The mean lower esophageal sphincter residual pressure was elevated at 43.7 mmHg. A calculated 69% of wet swallows were premature contractions with a distal latency value less than 4.5 seconds. The mean distal contractile integral was 13613 mmHg. This corroborates the spastic nature of type III achalasia.

**Conclusions:**

Considering that our patient developed anosmia and knowing its pathogenesis in the context of COVID-19, an inappropriate immune response to SARS-CoV-2 could have equally taken place in the esophagus. This inflammatory response might have caused the degeneration of the inhibitory neurons in the myenteric plexus responsible for the achalasia. Furthermore, given the fact that our patient had diarrhea, this suggests that the virus has a GI tropism supporting our hypothesis. We therefore believe that achalasia may be a potential complication of COVID-19.

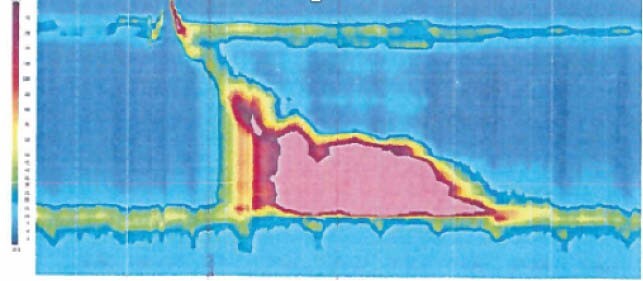

High resolution esophageal motility study showing a type III achalasia pattern.

**Funding Agencies:**

None

